# Explainable machine learning-based prediction of early and mid-term postoperative complications in adolescent tibial fractures

**DOI:** 10.3389/fsurg.2025.1688702

**Published:** 2025-10-21

**Authors:** Yufeng Wang, Jingxia Bian, Yang Yuan, Cong Li, Yang Liu

**Affiliations:** Shanghai Children’s Medical Center, Shanghai Jiao Tong University School of Medicine, Shanghai, China

**Keywords:** adolescent tibial fracture, postoperative complications, explainable machine learning, automated machine learning, clotting function, risk prediction, swarm intelligence optimization, clinical decision system

## Abstract

**Background:**

Adolescent tibial fractures commonly lead to postoperative complications. Conventional coagulation markers (PT/APTT/FIB) lack combinatorial risk assessment. We developed an explainable ML model integrating coagulation and clinical features to predict adverse events.

**Methods:**

A retrospective cohort of 624 surgical patients (13–18 years) was analyzed. AutoML with Improved Harmony Search Optimization (IHSO) processed features: age, fracture classification, surgery duration, blood loss, and 24 h-postoperative labs (coagulation triad/D-dimer/CRP). Primary outcome: 90-day composite adverse events (DVT/infection/early callus formation disorder/reoperation). SHAP explained predictions.

**Results:**

Baseline characteristics were balanced between training and test sets (*P* > 0.05). The IHSO-optimized algorithm outperformed controls in 91.67% of CEC2022 benchmark functions. AutoML model performance significantly surpassed conventional methods: training set ROC-AUC: 0.9667, test set ROC-AUC: 0.9247 (PR-AUC: 0.8350). Decision curves demonstrated clinical net benefit across 6%–99% risk thresholds. Key feature importance ranked as: age > operative duration > fibrinogen > fracture classification > APTT > CRP > BMI > D-dimer. SHAP analysis revealed: 1) Increasing age significantly attenuates the risk contribution of surgery duration; 2) FIB >4.0 g/L + elevated CRP indicated coagulation-inflammation cascade; 3) AO-C type fractures carried highest risk.

**Conclusion:**

This AutoML model, validated through explainability techniques, confirms the core predictive value of age, operative duration, and coagulation-inflammation networks for adolescent tibial fracture risk management. Though requiring prospective validation, the three-tier warning system establishes a stepped framework for individualized intervention. Future studies should advance multicenter collaborations integrating dynamic monitoring indicators to optimize clinical applicability.

## 1 Introduction

Adolescent tibial fractures represent one of the most common sports-related lower limb traumas in individuals aged 12–18 years, accounting for approximately 15%–25% of lower extremity fractures in this age group (1, 2). For such fractures, the early to mid-term postoperative period (typically within 3 months after surgery) is a critical phase for functional recovery. However, this process is frequently complicated by various adverse events. Clinically, deep vein thrombosis (DVT), surgical site infection (SSI), and impaired early callus formation are particularly notable complications, with significantly higher incidence rates in open fractures compared to closed fractures (2, 3). Notably, current pathophysiological studies suggest that DVT, SSI, and callus formation disorders are not isolated events but rather share a common underlying mechanism—sustained post-traumatic inflammation and hypercoagulability—exhibiting complex interactions (4–7). These complications not only substantially prolong hospital stays and increase healthcare costs but may also cause potential damage to the still-open growth plates of adolescents, adversely affecting long-term bone development and limb function. Therefore, systematic evaluation and early warning of these complications are critically important.

The 24-h postoperative period serves as a critical observation window for systemic stress response, during which coagulation parameters carry significant prognostic value. Currently, clinical practice relies on the conventional coagulation triad [prothrombin time (PT), activated partial thromboplastin time (APTT), and fibrinogen (FIB)] as a foundational assessment of clotting function, yet its clinical value is often confined to single-threshold warnings (8–10). The combined dynamic patterns of these indicators and their interactions with inflammation and traumatic stress remain underexplored for predicting comprehensive postoperative complications risks. Traditional logistic regression encounters significant challenges when modeling such complex relationships, as it fundamentally relies on manually constructed interaction terms. This approach faces two major limitations during feature space expansion: First, the inherent linear kernel structure fails to adequately represent high-order nonlinear associations between variables; second, the model becomes vulnerable to regression coefficient distortion caused by multicollinearity effects (11).

In recent years, explainable artificial intelligence (XAI) techniques have offered innovative solutions to these challenges (12). This study pioneers the integration of Automated Machine Learning (AutoML) with the Shapley value interpretability framework to address two core questions: (1) how to utilize early postoperative coagulation and inflammatory biomarkers (combined with injury severity and patient factors) to construct a high-accuracy prediction model for forecasting medium-to-early-term postoperative complications in adolescent tibial fracture patients, and (2) how to translate “black-box” predictions into actionable clinical insights. By establishing an integrated decision tool with both predictive power and clinical interpretability, this work enables personalized postoperative complications risk stratification for adolescent fracture patients, providing a theoretical foundation for early, precise intervention strategies.

## 2 Data and method

### 2.1 Study subjects

This study adopted a retrospective cohort design and consecutively enrolled 624 adolescent tibial fracture patients who underwent open reduction and internal fixation (ORIF) at a Grade III Class A orthopedic center from January 2019 to December 2023. Inclusion criteria: (1) age meeting the medical definition of adolescents (13–18 years); (2) closed unilateral tibial shaft or metaphyseal fracture confirmed by CT (excluding Salter-Harris type IV/V fractures involving the epiphysis); (3) fixation using a standardized locking compression plate (LCP) system; (4) availability of complete 90-day postoperative follow-up records. Exclusion criteria: (1) open fracture (Gustilo type II or higher); (2) concomitant craniocerebral or thoracoabdominal trauma requiring emergency surgery; (3) history of coagulation dysfunction (coagulation factor activity <50%) or use of anticoagulant/antiplatelet drugs within the past 3 months; (4) renal insufficiency (eGFR <60 ml/min/1.73m^2^) or liver disease (Child-Pugh ≥ B). In the final cohort, the training set (*n* = 499) and test set (*n* = 125) were divided in a 4:1 ratio through stratified random sampling to ensure consistent proportions of adverse prognostic events between the two groups. This study protocol was approved by the hospital ethics committee (Ethics Approval Number: IRB-2024-ortho038), and the research process strictly adhered to the Declaration of Helsinki and relevant norms for medical data management, ensuring full protection of patient rights and the legality and compliance of data collection, collation, and analysis.

### 2.2 Data collection and preprocessing

All data were collected through dual channels: the hospital electronic medical record (EMR) system and a specialized trauma database, encompassing three categories of core information: (1) baseline characteristics: Age, sex, body mass index (BMI), fracture AO/OTA classification (assessed blindly by two attending physicians), injury mechanism (sports injury/traffic accident/fall from height/other), surgical duration (from skin incision to suture completion), intraoperative blood loss (measured by suction and gauze weighing method). (2) Perioperative laboratory indicators: Routine coagulation triad (PT, APTT, FIB) and D-dimer (immunoturbidimetric method), CRP (latex-enhanced nephelometry) were collected under fasting conditions within 24 h postoperatively and detected using the Sysmex CS-5100 system. Anticoagulant tube specimens were centrifuged within 30 min (3,000 rpm × 15 min) and stored at −80 °C for testing. (3) Outcome measures: The primary outcome was composite medium-to-early-term postoperative complications adverse events occurring within 90 days postoperatively, defined as meeting at least one of the following conditions: Symptomatic deep vein thrombosis (DVT) or non-fatal pulmonary embolism (PE) confirmed by imaging or ultrasound; Confirmed surgical site infection (deep or superficial incision infection) requiring antibiotic treatment or surgical intervention; Standard radiographic evaluation reveals significantly insufficient or absent callus formation at the fracture site, manifested as clearly visible fracture lines without bridging callus formation; Internal fixation failure (e.g., screw loosening, plate fracture); Non-infectious wound complications requiring reoperation (e.g., poor healing, dehiscence, hematoma compression). Events were assessed and confirmed blindly by two senior orthopedic surgeons independent of this study, with disagreements resolved by a third expert.

Data processing followed three standardized procedures: (1) missing data handling: The overall missing rate was <3%, meeting conventional standards for low missing-rate datasets. For missing values in continuous variables, we employed multiple imputation with five imputations and used predictive mean matching algorithms to preserve reasonable data distributions. For missing categorical variables, mode imputation was applied to maintain category completeness. To further clarify the missing data mechanism, Little's test for missing completely at random (Little's MCAR test) was conducted, yielding a *p*-value >0.05, supporting the assumption that data were missing completely at random (MCAR)—meaning missingness was unrelated to both observed and unobserved variables. This validation reinforces the appropriateness of multiple imputation and effectively reduces potential selection bias due to missingness, particularly in analyses involving outcome measures such as adverse events, ensuring robust and reliable estimation results. (2) Sample balancing: To address the mild imbalance in the training set where adverse events accounted for 25.3% (126/499), the SMOTENC (Synthetic Minority Over-sampling Technique for Nominal and Continuous) algorithm was used to synthesize minority class samples, increasing adverse events to 40% (199/499) and retaining 70% of the original majority class samples (using a cluster cleaning mechanism to suppress noise generation). (3) Variable transformation: All continuous variables were standardized using *Z*-score; multicategorical data were one-hot encoded. (4) Data quality control: Through dual independent entry verification (Kappa consistency coefficient = 0.92) and logical checks for discrete variables. The complete feature list is provided in Appendix Table S1.

### 2.3 Prediction model construction

#### 2.3.1 Automated machine learning model

This study employed an automated machine learning (AutoML) model based on an optimization algorithm, deeply integrating a triple synergistic mechanism encompassing base learner selection, feature screening, and hyperparameter optimization. This framework unified three types of decision spaces into a mixed solution vector:

$$x=(\underset{\mathrm{model}\;\mathrm{type}}{\underbrace{k}}|\underset{\mathrm{feature}\;\mathrm{selection}}{\underbrace{\delta_{1},\delta_{2},\ldots ,\delta_{m}}}|\underset{\mathrm{hyperparameter}}{\underbrace{\lambda_{1},\lambda_{2},\ldots ,\lambda_{n}}})$$

where *k* is a discrete variable (1 = Logistic Regression, 2 = Support Vector Machine, 3 = Adaboost, 4 = XGBoost, 5 = LightGBM); the feature selection vector *δ* adopts 0/1 encoding; hyperparameters *λ* are dynamically adapted based on model characteristics.

The optimization process is driven by a swarm intelligence algorithm. Each evaluation executes core operations: first, determining the base learner type based on the *k*-value; then screening feature subsets via the *δ* vector; finally, injecting *λ* parameters into the model architecture. Configured models undergo comprehensive evaluation via 10-fold cross-validation, forming a synergistic feedback loop of “model architecture-feature representation-parameter configuration.” The core of collaborative optimization is the dynamically weighted fitness function:

$$f(x)=w_{1}(t)\cdot \mathrm{AC}C_{\mathrm{CV}}+w_{2}\cdot \left(1-\frac{{\left||\delta \right\|}_{0}}{m}\right)+w_{3}\cdot \exp(-T/T_{\max})$$

**Figure 1:**
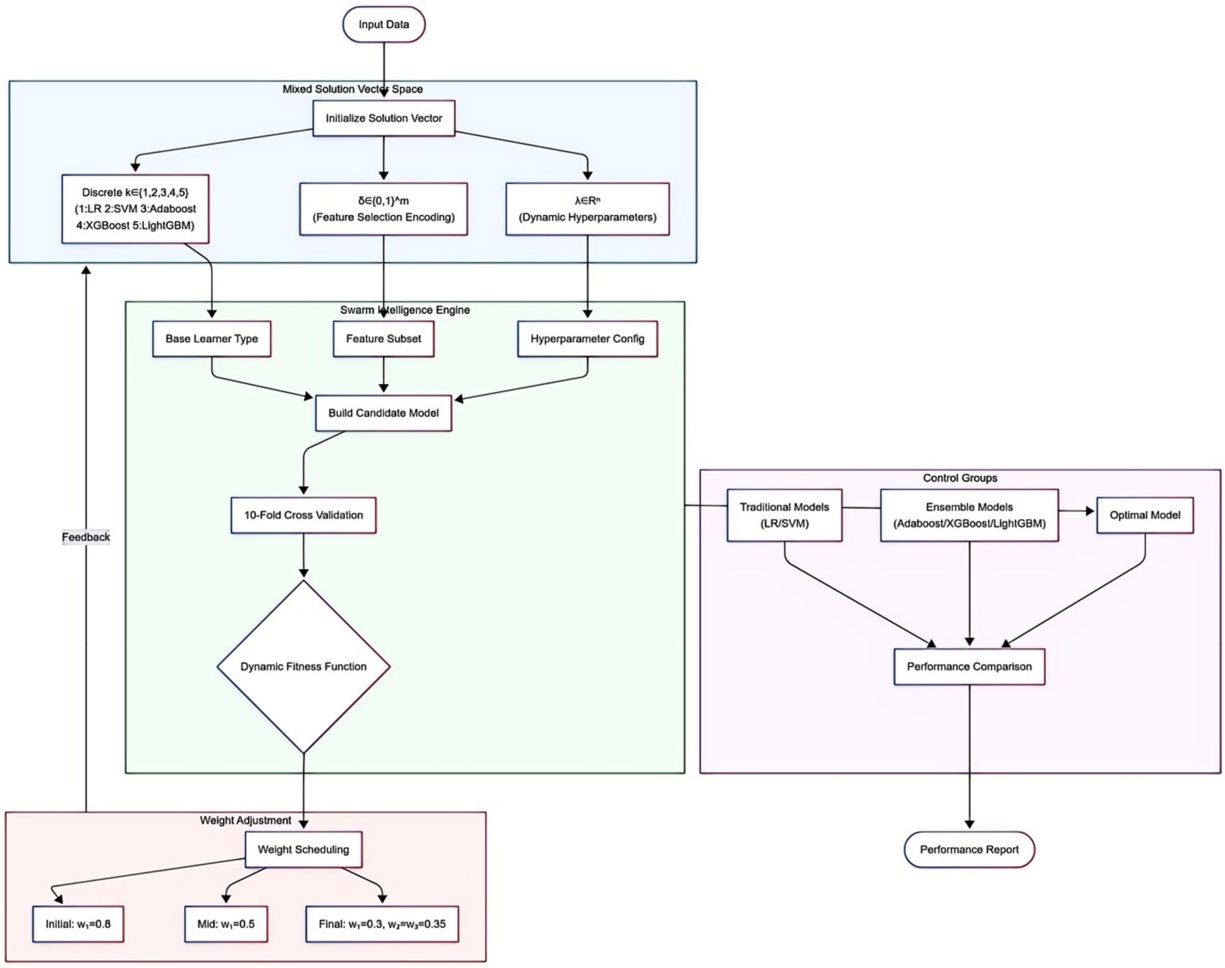
Flowchart of the automated machine learning model.

This function integrates three key dimensions: model accuracy (ACC term), feature sparsity, and time-computational cost (exponential decay term). Weight coefficients *w*_1_, *w*_2_, *w*_3_ are dynamically adjusted per iteration *t*: initial phases prioritize accuracy (*w*_1_ dominant); mid-phases balance accuracy and sparsity; final phases emphasize model simplification (*w*_1_ adjusted), with *w*_2_ and *w*_3_ set to equal weights. Traditional machine learning models (LR and SVM) and ensemble learning models (Adaboost, XGBoost, and LightGBM) were included for performance comparison. The AutoML flowchart is shown in Figure 1.

#### 2.3.2 Improved swarm intelligence algorithm method

This study employed the classical Holistic Swarm Optimization (HSO) (13) algorithm to guide AutoML optimization. HSO is a novel nature-metaphor-independent swarm intelligence optimization method. Unlike traditional algorithms that rely solely on individual experience or local neighborhoods for search decisions, HSO instead leverages the information distribution and fitness landscape of the entire population to guide optimization paths—akin to a comprehensively depicted “swarm map,” where each individual's actions are precisely adjusted within this panoramic framework. The algorithm intentionally eschews specific natural analogies or bio-inspiration in its design, focusing instead on rational mechanisms. It achieves a dynamic balance between global exploration and local exploitation through root-mean-square fitness-guided displacement coefficients, a selection process controlled by simulated annealing strategies, and an adaptive perturbation mechanism.

Building upon the original HSO, this study first reconstructs the initial population using chaotic mapping, enhancing search space diversity via nonlinear stochastic sequence generation strategies. Second, a dynamic spiral exploration mechanism is integrated, combined with Cauchy mutation perturbation to adjust the global-local convergence balance capability of individuals. This enhances the algorithm's adaptability to complex parameter spaces, ultimately yielding IHSO (Improved Holistic Swarm Optimization).

To further validate the performance of IHSO, the CEC2022 standard test functions were adopted to evaluate the algorithm (14). Comparative algorithms included the original HSO, Particle Swarm Optimization (PSO) (15), Harris Hawks Optimization (HHO) (16), and Whale Optimization Algorithm (WOA) (17). Twelve benchmark functions were selected, with the variable dimension of all test functions set to 10, population size to 30, and maximum iterations to 500. Each algorithm was independently run 30 times to ensure statistical reliability, with the results of these 30 runs ultimately compared. At the empirical data level, dual validation was conducted by constructing a clinical prognosis prediction model: Robustness testing was performed through artificially injecting progressive data disturbances (0%–15% noise combined with 0%–30% missing values); meanwhile, a feature selection module was designed to evaluate the model's complexity control capability.

#### 2.3.3 Model predictive performance evaluation metrics

This study constructed a composite evaluation system from three dimensions—classification performance, calibration performance, and clinical application—to systematically validate the comprehensive efficacy of the model in prognostic prediction tasks. The specific metrics include:

1. Classification Performance:

Basic Metrics: Accuracy (ACC), Sensitivity (SEN), and Specificity (SPE) quantify the model's overall discriminative power, ability to identify positive samples, and ability to exclude negative samples, respectively. Comprehensive Metric: The F1-score was used to evaluate the balance between precision and recall. Curve Evaluation: The Area Under the Receiver Operating Characteristic Curve (AUC-ROC) measures the model's ability to distinguish between different outcomes. The Area Under the Precision-Recall Curve (AUC-PR) evaluates the model's robustness in imbalanced sample scenarios. The DeLong test was employed to compare the statistical differences in AUC-ROC between different models.

1. Calibration Performance:

Calibration curves combined with the Brier score (lower values indicate greater prediction accuracy) were used to assess the accuracy of probability predictions.

1. Clinical Application:

This study employed Decision Curve Analysis (DCA) to quantify the clinical utility value of the prediction model. This method evaluates the clinical decision-making efficacy of the model across continuous risk thresholds by calculating the Net Benefit (NB), objectively measuring the benefit-risk balance of model-guided interventions. The calculation of Net Benefit integrates the benefit of correctly identifying true positive cases with the cost of misclassifying false positive cases. This method overcomes the limitation of traditional metrics in quantifying clinical utility, intuitively demonstrating the value of the model's intervention recommendations under different risk preferences and providing an empirical basis for personalized treatment.

### 2.4 Interpretability analysis

After preliminary feature screening for prognostic prediction via the AutoML framework, the study further employed LASSO regression analysis to validate the robustness of the selected features. Finally, the SHAP interpretability model was utilized to analyze the clinical rationality of these features. The specific workflow is as follows:

1. AutoML Initial Feature Screening:

Based on predefined search spaces and optimization objectives, AutoML algorithms automatically identified the subset of features significantly associated with prognosis.

1. LASSO Feature Validation:

LASSO regression was applied to the feature subset screened by AutoML. The sparsity and stability of these features were validated through a regularization constraint mechanism, ensuring the key features' resistance to overfitting. Differences between features selected by LASSO and those automatically screened by AutoML were compared.

1. SHAP (Shapley Additive Explanations) Interpretability Analysis:

The SHAP algorithm, constructed based on game theory, quantified the contribution of model features. Global feature importance rankings revealed the overall impact strength of key variables, enabling visualization of the prediction logic and thereby validating its rationality.

### 2.5 Statistical analysis

The research data were uniformly imported into the SPSS26.0 statistical analysis platform for standardized processing. Continuous variables conforming to normal distribution were expressed as mean ± standard deviation (*x* ± *s*); continuous variables not conforming to normal distribution were expressed as median (interquartile range) [M (P25, P75)]; categorical variables were expressed as frequency and percentage [*n*(%)]. For intergroup comparisons, for continuous variables, normality tests were first performed. If both groups of data conformed to normal distribution, a one-way *t*-test was used for intergroup comparison; if the data did not conform to normal distribution, the Mann–Whitney *U* test was used for intergroup comparison. For categorical variables, intergroup comparison was performed using Pearson's chi-square test. The test efficacy was based on the *P*-value (two-sided test, significance threshold set to *α* = 0.05). The research results were presented in a structured tabular format.

## 3 Result

### 3.1 Comparison of baseline data

The average age of the overall sample was (15.64 ± 1.78) years, comprising 402 males and 222 females. The training set (*n* = 499) and the test set (*n* = 125) showed no statistically significant differences in all baseline characteristics and laboratory indicators (all *P* > 0.05), indicating effective random stratified sampling: the proportions of poor prognosis events were highly consistent between the two groups (training set 25.25% vs. test set 25.60%, *χ*^2^ = 0.006, *P* = 0.936). Details are presented in Table 1.

**Table 1:** Comparison of baseline characteristics between training and test sets.

Feature	Training set	Test set	Statistic	*P-value*
	(*n* = 499)	(*n* = 125)		
Poor outcome events, *n* (%)	126 (25.25%)	32 (25.60%)	*χ*^2^ = 0.006	0.936
Age (years)	15.42 ± 1.75	15.64 ± 1.87	*t* = 1.240	0.216
*(Mean* *±* *SD)*				
Male sex, *n* (%)	322 (64.53%)	80 (64.00%)	*χ*^2^ = 0.012	0.912
BMI (kg/m^2^)	20.35 [18.15–22.71]	20.49 [18.33–22.92]	*U* = 29,608	0.425
*[Median (IQR)]*				
AO/OTA Fracture Classification, *n* (%)			*χ*^2^ = 0.030	0.985
Type A (Simple)	177 (35.47%)	44 (35.20%)	—	—
Type B (Wedge)	198 (39.68%)	49 (39.20%)	—	—
Type C (Complex)	124 (24.85%)	32 (25.60%)	—	—
Injury mechanism, *n* (%)			*χ*^2^ = 0.165	0.983
Sports injury	211 (42.28%)	52 (41.60%)	—	—
Traffic accident	197 (39.48%)	49 (39.20%)	—	—
Fall from height (≥3 m)	60 (12.02%)	15 (12.00%)	—	—
Other	31 (5.61%)	9 (6.40%)	—	—
Surgery duration (min)	110 [87–138]	115 [90–143]	*U* = 29,521	0.44
*[Median (IQR)]*				
Blood loss (ml)	148 [107–193]	155 [115–200]	*U* = 29,650	0.352
*[Median (IQR)]*				
Postoperative lab parameters				
PT (s), Mean ± SD	12.55 ± 1.00	12.58 ± 1.07	*t* = 0.295	0.768
APTT (s), Mean ± SD	33.42 ± 3.45	33.51 ± 3.57	*t* = 0.259	0.795
FIB (g/L), Mean ± SD	3.63 ± 0.85	3.60 ± 0.92	*t* = 0.347	0.729
D-dimer (mg/L FEU)	0.85 [0.61–1.18]	0.87 [0.63–1.21]	*U* = 29,143	0.583
*[Median (IQR)]*				
CRP (mg/L)	19.35 [13.25–26.85]	20.15 [13.88–27.58]	*U* = 28,897	0.681
*[Median (IQR)]*				

SD, standard deviation; IQR, interquartile range; AO/OTA, Arbeitsgemeinschaft für Osteosynthesefragen/Orthopaedic Trauma Association; FET, Fisher's exact test; PT, prothrombin time; APTT, activated partial thromboplastin time; FIB, fibrinogen; FEU, fibrinogen equivalent units; CRP, C-reactive protein.

### 3.2 Improved algorithm simulation test

#### 3.2.1 Standard test function simulation experiments

**Figure 2:**
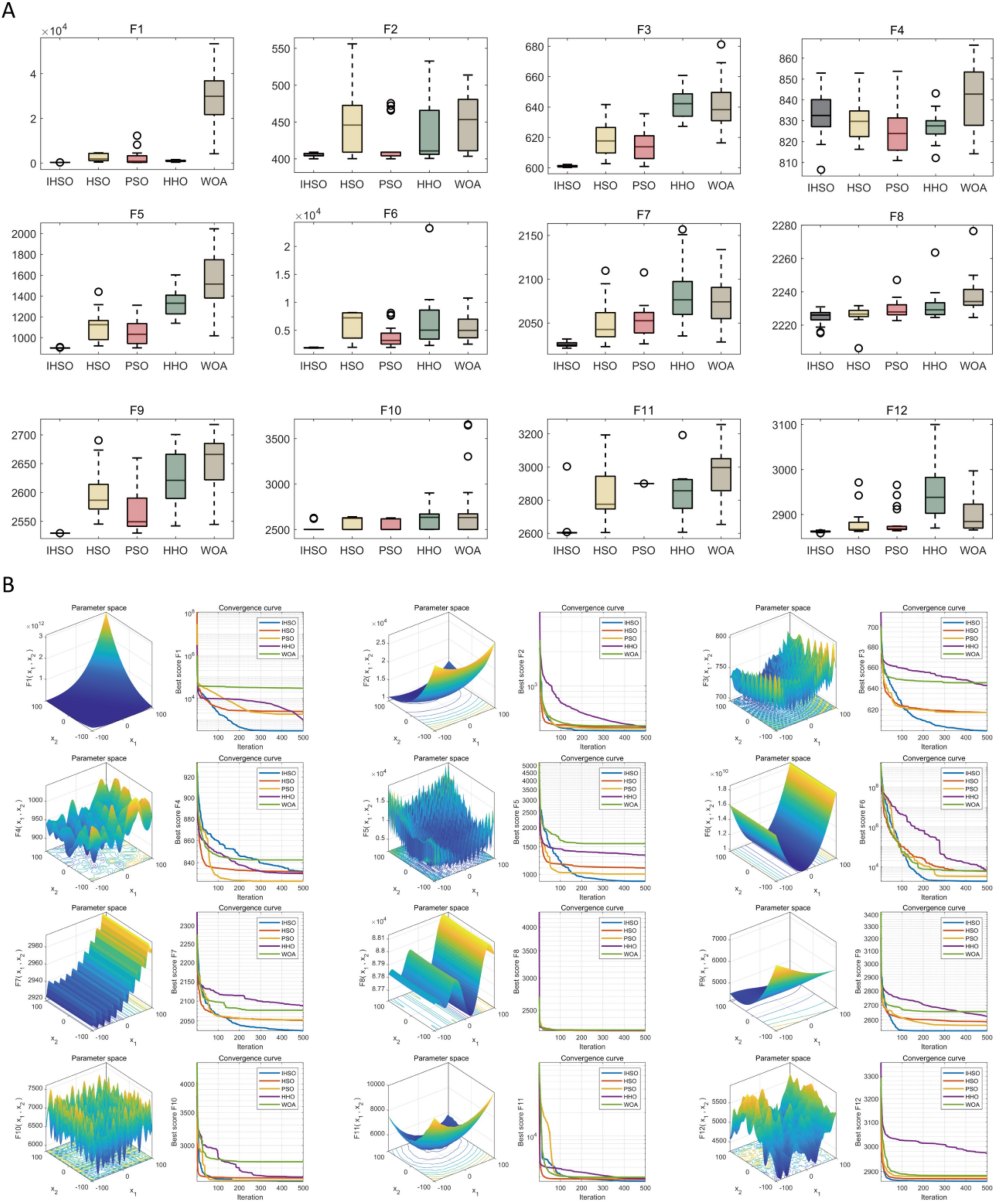
Performance simulation test of swarm intelligence algorithm optimization. **(A)** Box plots of optimization results after 30 independent runs of each algorithm on CEC2022 test functions, demonstrating the optimization stability and robustness of each algorithm; **(B)** convergence curves of each algorithm during the optimization process, reflecting their convergence speed and ability to avoid local optima.

Based on a standardized evaluation framework, this study systematically compared the improved IHSO algorithm with the original HSO and other swarm intelligence algorithms (PSO, HHO, WOA) across 12 benchmark functions of the CEC2022 test system. Statistical robustness was verified through 30 independent repeated experiments. The results demonstrated that the improved algorithm achieved optimal solutions in 11 functions (91.67%) (Figure 2A). Its solution distribution range exhibited significant convergence with fewer outliers, indicating that the proposed mechanism effectively enhanced optimization stability. Dynamic iteration analysis (Figure 2B) revealed that IHSO achieved the fastest convergence speed in key test functions (F1, F3, F5, F6, F7, F9, and F10), proving its comprehensive improvement in balancing global exploration (rapid early-stage decline) and local exploitation (fine-tuned late-stage optimization) capabilities.

#### 3.2.2 Analysis of real-world clinical data

**Figure 3:**
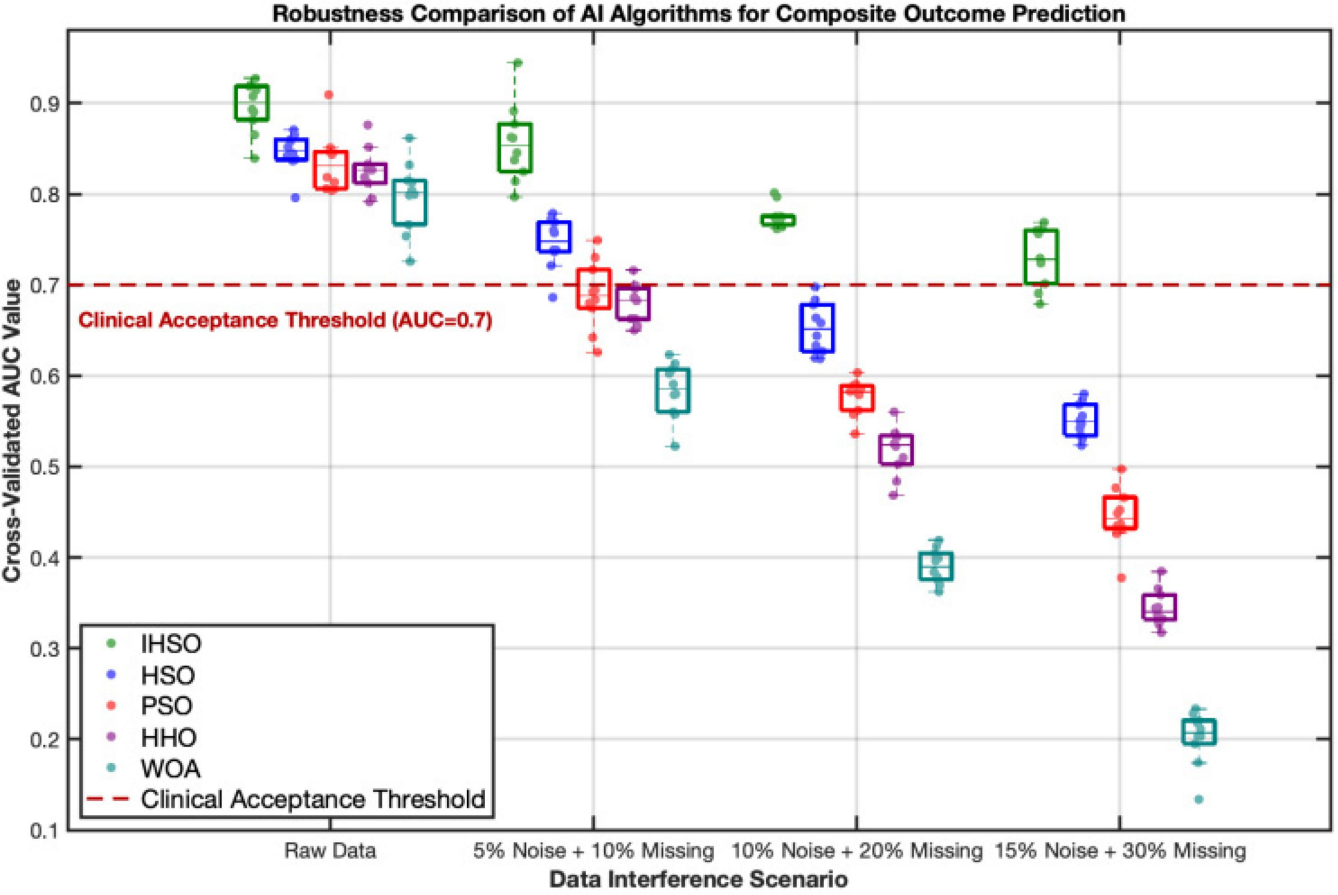
Robustness comparison of intelligent algorithms in predicting composite postoperative outcomes. This figure demonstrates the stability performance of five intelligent algorithms (IHSO/HSO/PSO/HHO/WOA) under increasing data interference scenarios. The boxplots represent the distribution of cross-validated AUC values from 10 repeated experiments, while the scatter points indicate single experimental results. Algorithm color coding: IHSO (forest green), HSO (blue), PSO (red), HHO (purple), WOA (cyan). The red dashed line indicates the clinically acceptable threshold (AUC = 0.7), values below which are considered insufficient for clinical reference.

By artificially injecting progressive data perturbations, this study evaluated the performance of prognostic prediction models based on real-world clinical data. Experimental results demonstrated that all intelligent algorithms exhibited performance degradation trends as perturbation intensity increased (Figure 3). Under original data conditions, the five types of algorithms achieved an average prediction accuracy of AUC = 0.84; however, under extreme perturbation scenarios (15% noise combined with 30% missing values), model performance significantly declined to an average AUC = 0.71, representing a relative decrease of 15.5%. Among them, the IHSO algorithm displayed optimal stability: its prediction accuracy decreased from AUC = 0.91 in the original scenario to AUC = 0.78 in high-perturbation scenarios, with a relative decline (14.3%) significantly lower than other algorithm groups (all declines >20%).

**Figure 4:**
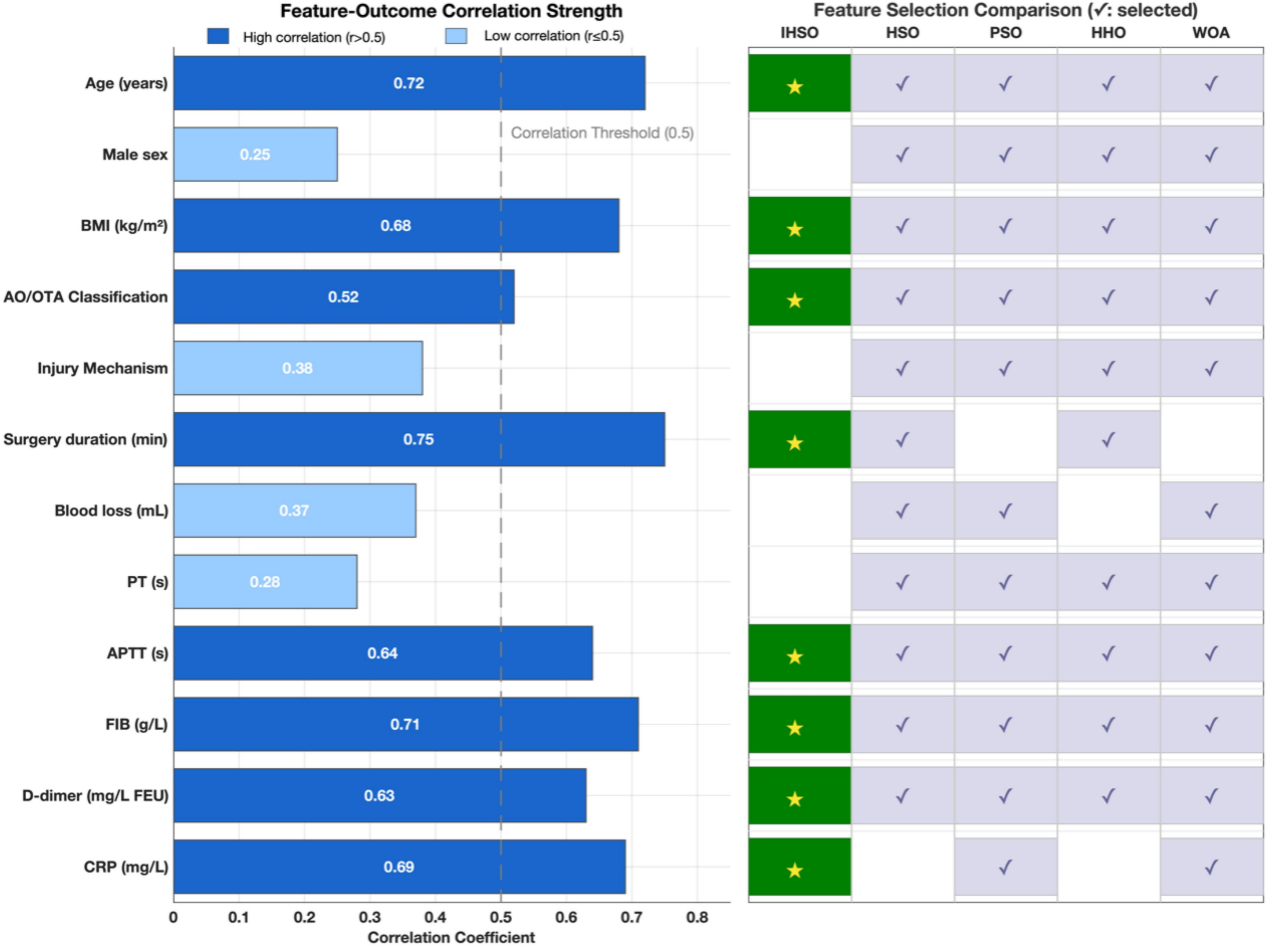
Clinical feature analysis and feature selection optimization. This study compared the correlation strength between 12 clinical features and postoperative outcomes, and evaluated the feature selection performance of the IHSO algorithm against other optimization algorithms (HSO, PSO, HHO, WOA). The left panel displays the correlation coefficients (*r*) between each feature and the outcomes, with dark blue indicating highly correlated features (*r* > 0.5), light blue indicating weakly correlated features (*r* ≤ 0.5), and the gray dashed line representing the correlation threshold of 0.5. The right panel shows the feature selection results of the algorithms, where dark green squares (★) represent features selected by IHSO, and light blue squares (✓) represent features selected by other algorithms.

At the key feature identification level, the IHSO algorithm demonstrated excellent compactness characteristics (Figure 4). It stably screened out 8 highly correlated predictors (average correlation coefficient *r* = 0.67, SD = 0.08), representing a 26% reduction compared to the average of 10.8 features selected by other algorithms. This feature refinement mechanism effectively reduced model complexity while maintaining prediction accuracy.

### 3.3 Training set cross-validation comparison

**Figure 5:**
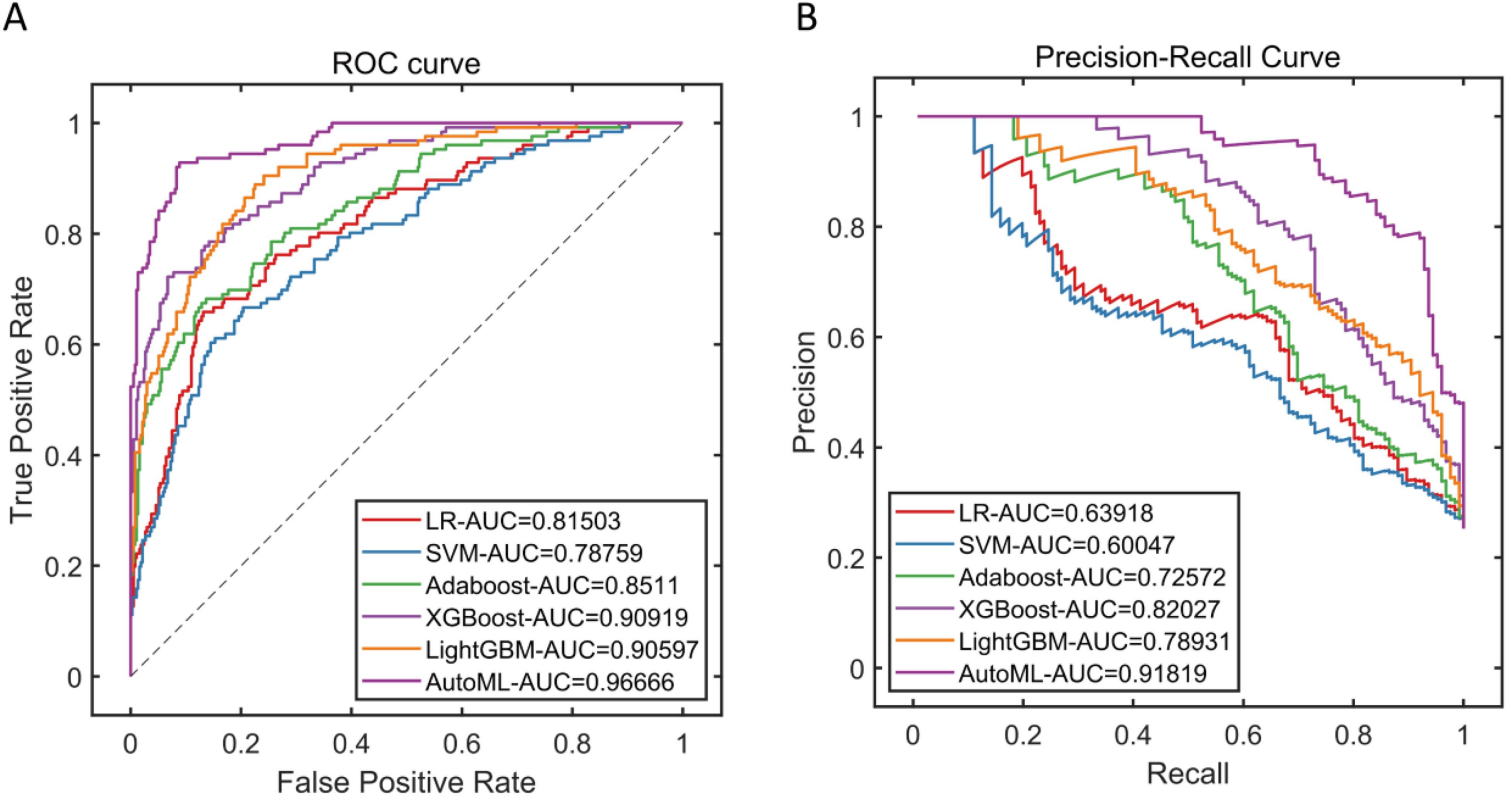
Performance of training Set cross-validation. **(A)** Training set ROC curve; **(B)** Training set PR curve.

This study systematically evaluated the prediction performance of six machine learning models on the training set, including metrics such as precision, sensitivity, specificity, accuracy, F1-score, and area under the curve. The results demonstrated that the AutoML model exhibited comprehensively optimal performance, with its ROC-AUC reaching 0.9667 and PR-AUC at 0.9182. Notably, AutoML showed particularly outstanding advantages in the F1-score (0.8928), indicating its stronger clinical application value in balancing precision and recall. The features ultimately selected by AutoML were: age, surgery duration, FIB, AO/OTA, APTT, CRP, BMI, and D-dimer. Details are provided in Table 2 and Figure 5.

**Table 2:** Prediction performance metrics of training set cross-validation.

Model	PRE	SEN	SPE	ACC	F1	ROC-AUC	PR-AUC
LR	0.3181	0.9365	0.3217	0.4770	0.4748	0.8150	0.6392
SVM	0.2725	0.9841	0.1126	0.3327	0.4269	0.7859	0.6005
Adaboost	0.3833	0.9127	0.5040	0.6072	0.5399	0.8511	0.7257
XGBoost	0.4754	0.9206	0.6568	0.7234	0.6270	0.9092	0.8203
LightGBM	0.3761	0.9762	0.4531	0.5852	0.5430	0.9060	0.7893
AutoML	0.7452	0.9286	0.8928	0.9018	0.8269	0.9667	0.9182

### 3.4 Test set prediction performance comparison

**Figure 6:**
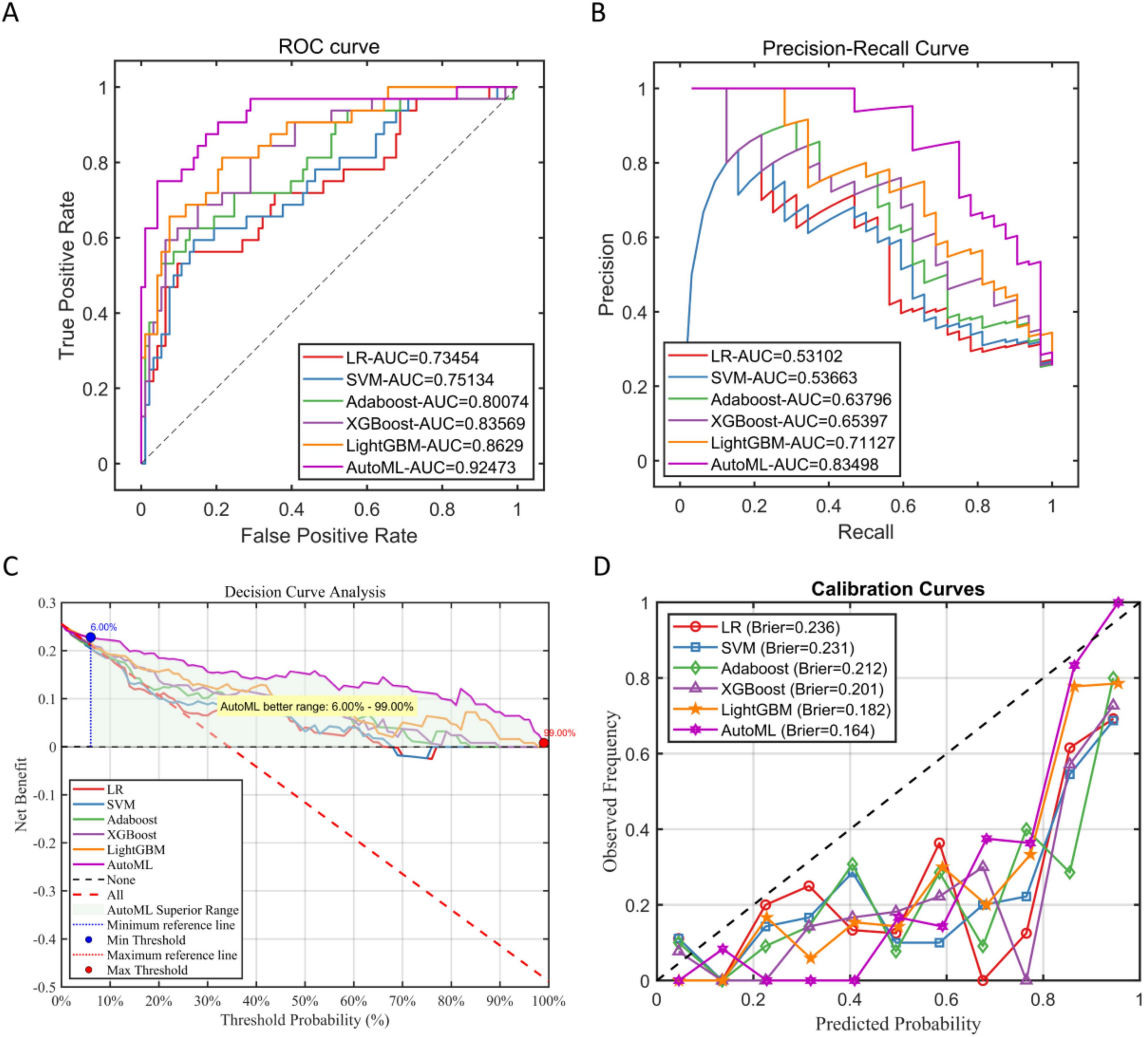
Performance of test Set. **(A)** Test set ROC curve; **(B)** test set PR curve; **(C)** test set DCA curve; **(D)** test set calibration curve.

In this study, in the prediction task of medium-to-early-term postoperative complications adverse events after adolescent tibial fracture surgery, the performance of six machine learning models on the test set was systematically evaluated. The results showed that AutoML exhibited the strongest robustness in the independent test set, with ROC-AUC reaching 0.9247 and PR-AUC at 0.8350 (Figure A,B); Decision curve analysis (Figure 3C) showed that applying the AutoML prediction model in the test set within the risk threshold range of 6% to 99% could bring greater clinical net benefit compared to traditional methods; the net benefit curve of this model could maintain a high level and remain stable over a wide range of threshold probabilities, indicating its good generalization ability and stable prediction performance; Calibration curve analysis (Figure 3D) confirmed that the predictive calibration performance of the AutoML model was significantly better than other models, with its test set Brier score (0.164) being the lowest. Details are provided in Table 3 and Figure 6.

**Table 3:** Prediction performance metrics of test set.

Model	PRE	SEN	SPE	ACC	F1	ROC-AUC	PR-AUC
LR	0.2672	0.9688	0.0860	0.3120	0.4189	0.7345	0.5310
SVM	0.2581	1.0000	0.0108	0.2640	0.4103	0.7513	0.5366
Adaboost	0.3229	0.9688	0.3011	0.4720	0.4844	0.8007	0.6380
XGBoost	0.3131	0.9688	0.2688	0.4480	0.4733	0.8357	0.6540
LightGBM	0.4906	0.8125	0.7097	0.7360	0.6118	0.8629	0.7113
AutoML	0.5357	0.9375	0.7204	0.7760	0.6818	0.9247	0.8350

### 3.5 Subgroup analysis

**Figure 7:**
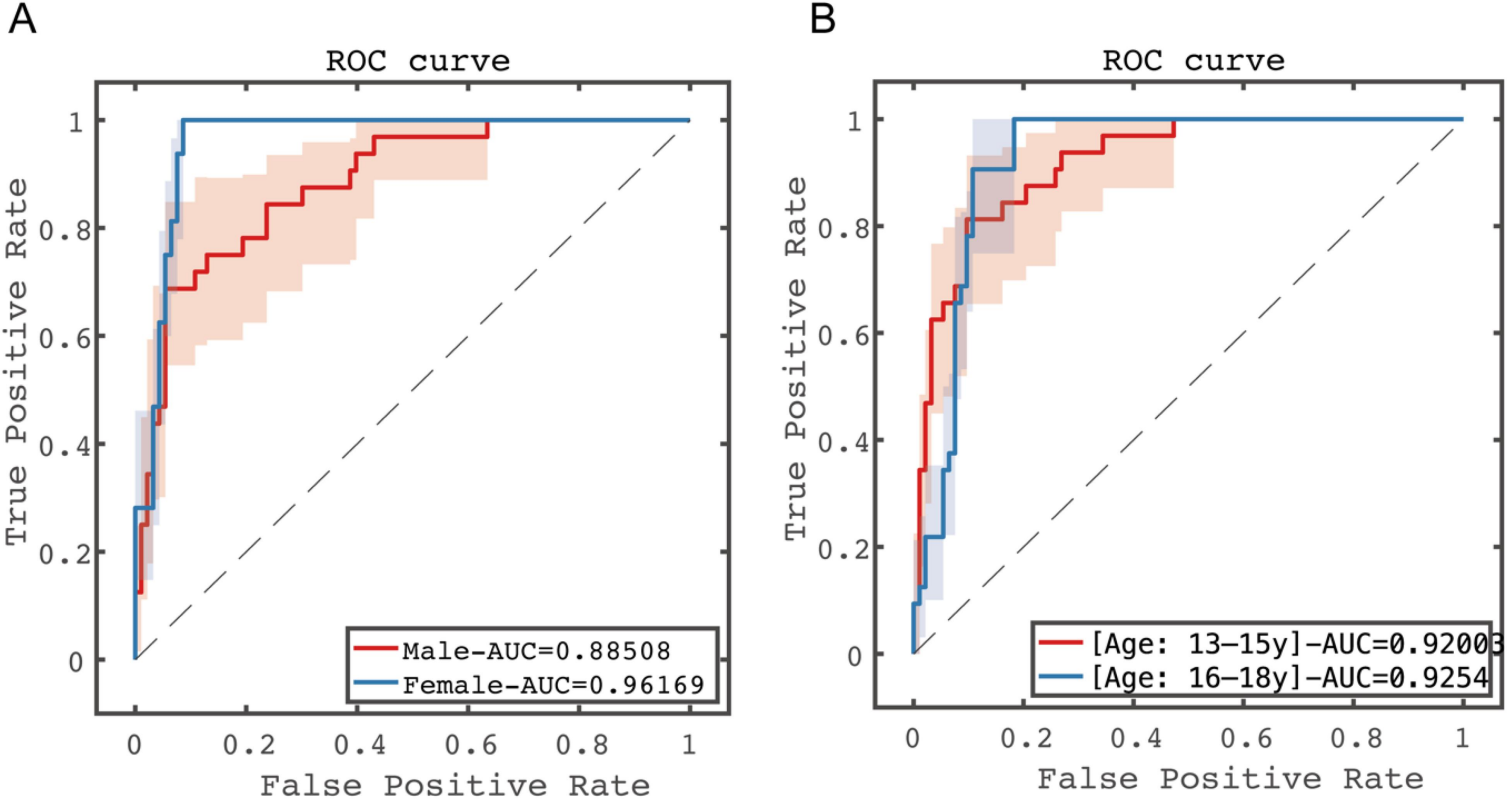
Predictive performance results of subgroup analysis in the test set. **(A)** Gender subgroup analysis; **(B)** age subgroup analysis.

Stratified analyses were conducted by gender (male and female) and age (divided into two subgroups: 13–15 years and 16–18 years). The results showed significant differences among gender subgroups, with female patients demonstrating significantly better prognostic prediction performance than males (AUC: 0.8851 for females vs. 0.9617 for males, *p* < 0.05). No statistically significant difference was observed between age subgroups (AUC: 0.9200 for 13–15 years vs. 0.9254 for 16–18 years, *p* > 0.05). See Figure 7 for details.

### 3.6 Interpretability analysis

#### 3.6.1 Feature validation

**Figure 8:**
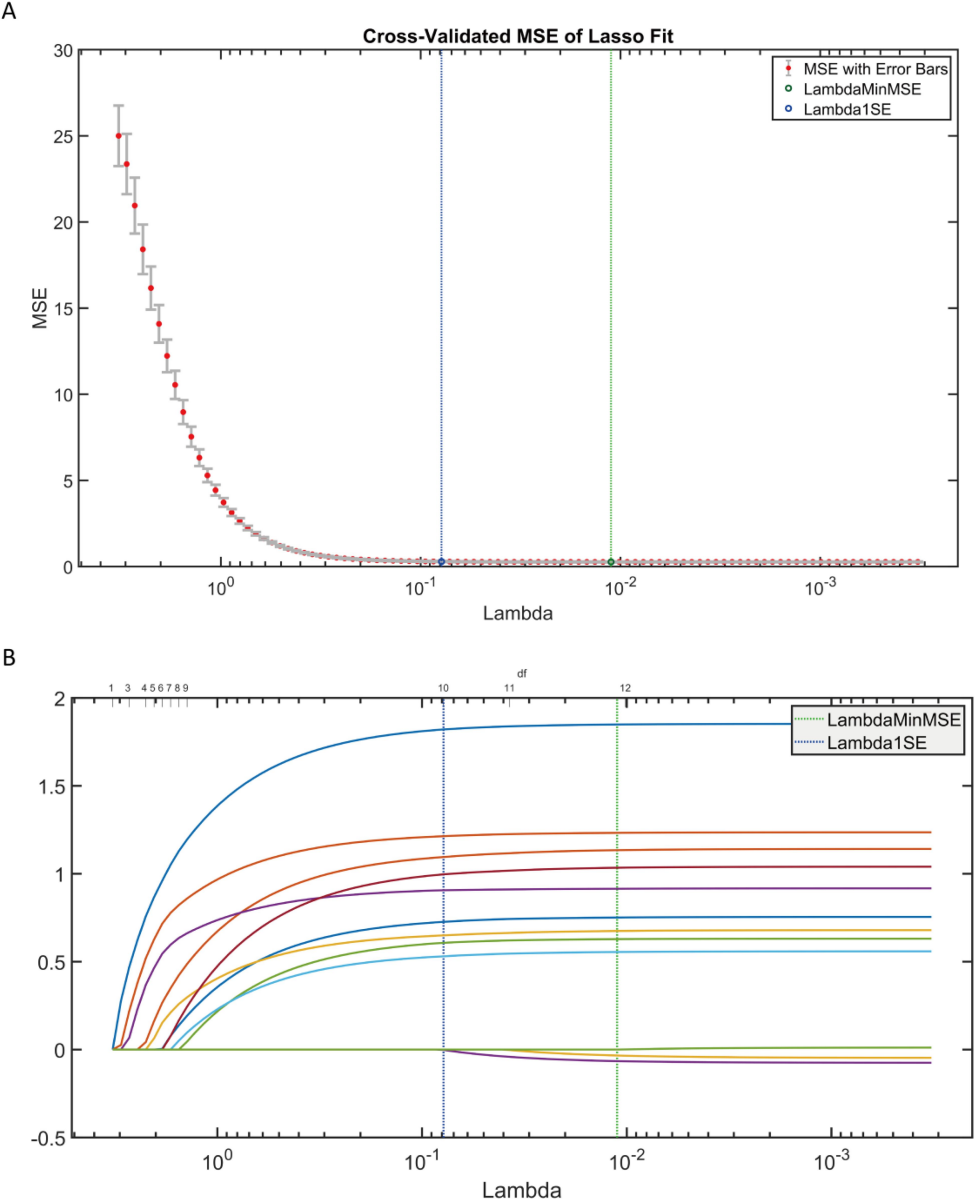
LASSO regression results. **(A)** LASSO trajectory plot; **(B)** LASSO cross-validation fitting plot.

Adopted LASSO regression for feature screening on the training set data (Figure 8), to validate the effectiveness of the AutoML model in feature screening. LASSO selected variables within one standard error of the minimum MSE in the sparse model (Lambda1SE), screened out 9 variables: age, surgery time, FIB, AO/OTA, APTT, CRP, BMI, D-dimer, blood loss, with an overlap rate with AutoML screened features of 88.89% (8/9).

#### 3.6.2 SHAP analysis

**Figure 9:**
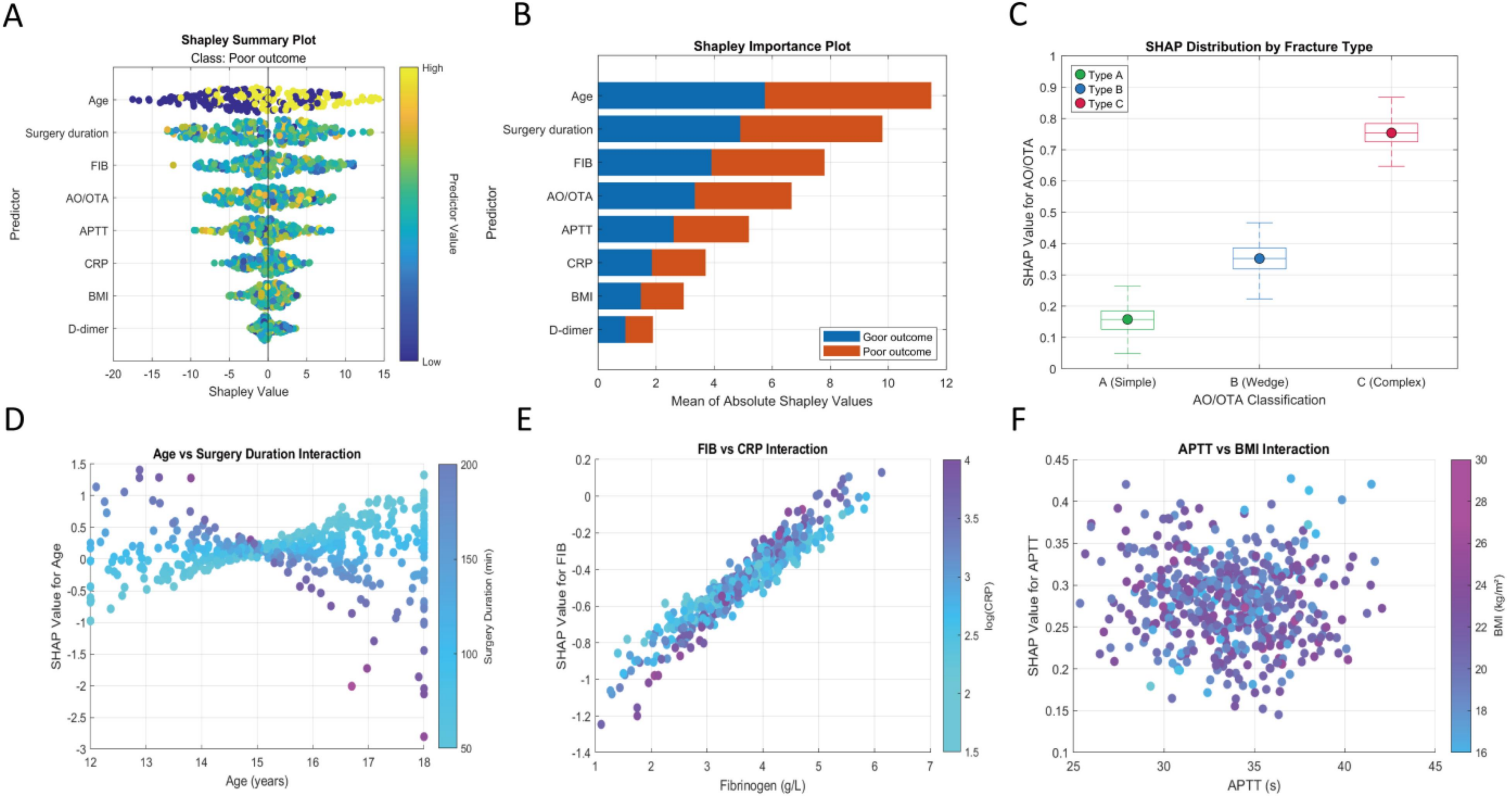
Machine learning interpretability analysis. **(A)** Shapley summary plot; **(B)** shapley feature importance plot; **(C)** SHAP values for different AO/OTA classifications; **(D)** SHAP interaction plot for age and surgery duration; **(E)** SHAP interaction plot for fibrinogen and C-reactive protein; **(F)** SHAP interaction plot for APTT and BMI.

According to the SHAP analysis results in Figure 6, the importance ranking is: age, surgery time, FIB, AO/OTA, APPT, CRP, BMI, D-dimer (Figures 9A,B); the baseline risk of complex fractures is higher than that of simple fractures (Figure 9C); the interaction effect between age and surgery duration (Figure 9D) shows that increasing age significantly attenuates the risk contribution of surgery duration. This is manifested as a concentrated negative shift in the SHAP values of the age variable for older age groups (>15 years) undergoing prolonged surgeries (>130 min), with the lowest value reaching −0.3; fibrinogen (FIB) > 4.0 g/L, along with elevated C-reactive protein (CRP), suggests the presence of a coagulation-inflammation cascade (Figure 9E); interaction between APTT and BMI (Figure 9F), a median activated partial thromboplastin time (APTT) (33–36 s) combined with a higher body mass index (BMI) (>22 kg/m^2^) indicates an increased risk of thrombosis.

### 3.7 Clinical decision system

**Figure 10:**
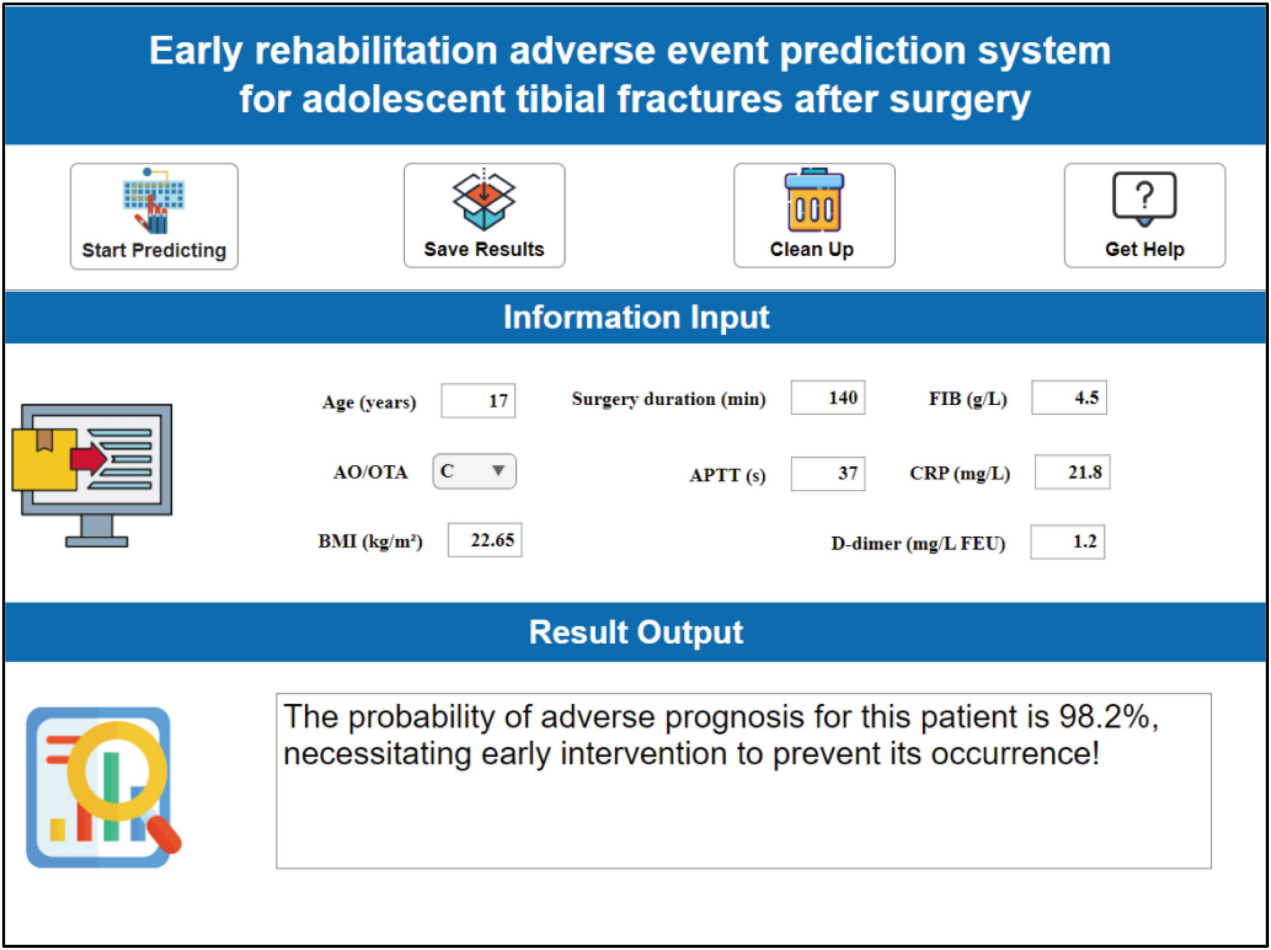
Clinical decision system for early postoperative rehabilitation adverse events in adolescent tibial fractures.

This system designs a visual and interactive prediction interface. After clinicians input key indicators such as the patient's age, surgery duration, fibrinogen (FIB) level, and fracture AO classification in the “Feature Input” panel, the system calculates the probability (0%–100%) of medium-to-early-term postoperative complications adverse events in real-time based on the trained AutoML model (Figure 10).

This risk probability is visually presented through a three-tier warning system: low risk (<30%) recommends routine rehabilitation follow-up; moderate risk (30%–70%) initiates an enhanced monitoring scheme; high risk (>70%) requires immediate intervention. For different risk levels, distinct clinical intervention pathways can be adopted: (1) low-risk patients: Implement a standard rehabilitation plan (outpatient follow-up at postoperative weeks 1, 2, and 4), with recommended basic anticoagulation measures and early weight-bearing training (progressively reaching 50% body weight load within 4 weeks postoperatively); (2) moderate-risk patients: Increase vascular ultrasound monitoring to twice weekly (to exclude DVT), elevate physical therapy frequency to every other day (including pulsed electromagnetic field stimulation), and upgrade pharmacological intervention (rivaroxaban + low molecular weight heparin bridging); (3) high-risk patients: Initiate multidisciplinary consultation (orthopedics/hematology/rehabilitation) within 48 h, enforce inpatient monitoring for ≥7 days, adjust drug regimen to therapeutic anticoagulation dose (enoxaparin 1 mg/kg bid), and implant a remote bone healing monitoring sensor (transmitting callus CT value changes daily).

## 4 Discussion

This study addresses the clinical needs for postoperative management of adolescent tibial fractures by constructing an integrated risk prediction system based on explainable machine learning. By integrating the Improved Harmony Search Optimization (IHSO) algorithm with an automated machine learning (AutoML) framework, the model systematically balances prediction accuracy and clinical interpretability. The eight key predictors selected by AutoML have clear clinical pathological foundations: age and surgery duration serve as primary drivers, reflecting the special sensitivity of adolescents in the skeletal development stage to surgical trauma; fibrinogen (FIB) surpasses traditional PT/APTT indicators in core importance, highlighting its “double-edged sword” characteristic—acting as both a substrate for thrombosis and a medium for tissue repair (8, 18); the FIB-CRP interaction effect, visualized through SHAP plots, confirms the pathological hub role of the coagulation-inflammation cascade (19–21); and the AO classification (especially type C complex fractures) serves as an objective measure of anatomical injury severity through mechanisms such as periosteal blood supply disruption and mechanical instability (22). This combination of features overcomes the limitations of single-indicator threshold warnings, achieving a holistic assessment of multidimensional pathological networks. Additionally, it was noted that postoperative normalization of D-dimer levels could serve as a biological marker of surgical success, with its prognostic value potentially deriving from its ability to quantify postoperative recovery capacity following surgical stress.

Regarding outcome measures, we adopted a pathophysiology-driven approach, considering that systemic stress responses in adolescents following tibial fractures exhibit distinct features compared to adults, with more pronounced crosstalk among coagulation, inflammation, and bone metabolism networks (5, 23). Multiple preclinical studies support this notion, suggesting that deep vein thrombosis (DVT), surgical site infection (SSI), and impaired early callus formation may share common driving pathways. For instance, persistently activated thrombin–protease-activated receptor (PAR) signaling post-trauma has been identified as a key integrating factor: upregulation of this pathway not only promotes a hypercoagulable state (potentially increasing DVT risk) but also exacerbates local inflammation and tissue damage through neutrophil extracellular trap (NET) release (linked to SSI risk), while simultaneously disrupting osteogenic signal transduction during early callus formation (affecting the initial healing process) (4, 5, 7). This pleiotropic mechanism implies that DVT, SSI, and impaired bone healing are not isolated events but rather different clinical manifestations potentially stemming from the same pathophysiological axis. Therefore, given this shared biological foundation, developing an integrated model capable of concurrently predicting the risk of multiple complications is rationally justified (6, 24). From a clinical decision-making perspective, the application value of composite endpoints lies in providing a comprehensive risk assessment tool that assists physicians in early identification of high-risk patients, thereby enabling targeted preventive strategies and optimized postoperative management. For example, in high-risk adolescent tibial fracture cases, the composite endpoint could screen for individuals with elevated multisystem complication risks, facilitating multidisciplinary interventions. Furthermore, this model is adaptable for postoperative monitoring, such as integrating multiple indicators during routine follow-ups to enhance both the timeliness and comprehensiveness of interventions. In summary, the composite endpoint design not only strengthens the biological plausibility of predictive models but also improves their clinical applicability and generalizability.

At the methodological level, the improved IHSO algorithm significantly enhances the stability and efficiency of the feature selection process through chaotic mapping initialization and dynamic spiral exploration mechanisms. More importantly, this study deeply integrates the game-theoretic interpretability framework (SHAP) into the AutoML workflow, generating three innovative values: first, the global feature importance ranking provides physicians with decision priority references, such as the higher regulatory value of surgery duration over BMI; second, the interaction effect plot reveals that in adolescents over 15 years old, the increase in age significantly reduces the contribution intensity of surgery duration to risk, which may be related to the enhancement of surgical stress compensation ability after growth plate closure (25, 26); third, the APTT-BMI “paradox effect” (where normal APTT becomes a risk factor in high-BMI populations) provides clinical evidence for the theory that adipokines interfere with coagulation balance (27, 28). The model maintains excellent performance on an independent test set, and decision curve analysis shows clinical net benefit across a 6%–99% risk threshold range, demonstrating the practical value of this computational pathology approach. However, compared to other orthopedic prediction models, this study adopts a composite endpoint design that more closely aligns with real clinical scenarios—covering synergistic pathological processes such as DVT, SSI, and delayed healing—avoiding the one-sidedness of predicting single complications.

In terms of population inclusion, the adolescent age definition (13–18 years) serves as the anatomical basis for model construction, with this standard calibrated by three pieces of evidence: developmental anatomy confirms that this stage covers the critical period of tibial growth plate closure, where growth plates in Sauvegard stage III–IV are highly sensitive to traction injuries; epidemiological data show that 14–17 years is the peak age for tibial shaft fractures, coinciding with high incidences of sports and traffic injuries; the boundary consideration also excludes pathological fracture patterns in those under 13 years (such as abuse fractures) and groups over 18 years who are suitable for adult internal fixation protocols (29, 30). However, this rigid division may overlook the specificity of individuals in the rapid growth period at 12–13 years, manifesting as a discontinuity in the risk spectrum for growth plate injuries, and future research needs to explore boundary effects.

In subgroup analyses, we stratified patients by gender and age to evaluate the predictive performance of the AutoML model across different populations. The results showed significant differences in gender subgroups but no notable variations in age subgroups. Specifically, the model demonstrated higher predictive sensitivity among female patients, which may be related to fluctuations in estrogen levels during adolescence. These differential results highlight the model's applicability in personalized medicine—for instance, clinical implementation could prioritize gender-specific risk-adjusted monitoring strategies, whereas the homogeneity in age factors supports the model's broader suitability for this adolescent population. Future research should further integrate biomarkers such as hormone levels to refine predictive accuracy for gender subgroups.

It is also crucial to clearly recognize the multiple limitations of this study: as a single-center retrospective study, although the 624 samples were validated for balance through stratified random sampling, selection bias cannot be overcome; population characteristics are concentrated in tertiary hospitals, not incorporating diagnostic and therapeutic variations in primary care settings; the decision system was only tested in a simulated environment, and its real-world effectiveness needs to be verified through pragmatic clinical trials with a stepped-wedge design; the lack of dynamic monitoring dimensions is particularly critical—postoperative changes in coagulation indicators within 72 h (such as the peak slope of D-dimer) were not captured, potentially missing important early warning signals of compensatory hypercoagulability; individual differences in anticoagulant drug metabolism (such as CYP2C9 gene polymorphisms) were also not included in the current model.

The future research optimization path should focus on four dimensions: (1) the primary task is to conduct prospective multicenter validation, with emphasis on monitoring the clinical compliance and misjudgment costs of the decision system in real workflows (establishing a traceability mechanism for missed adverse event reporting). (2) Building upon this, developing dynamic monitoring modules requires integrating minimally invasive sensing technologies to collect localized perfusion parameters such as tissue oxygen saturation and intraosseous pressure in real time. When selecting minimally invasive sensing devices, priority should be given to those with high biocompatibility, moderate sampling frequency (e.g., 1–5 times per min), and compliance with medical device safety standards to ensure patient safety and data reliability. Simultaneously, dynamic data must seamlessly interface with existing electronic health record systems through standardized protocols (such as HL7 or FHIR) to enable automatic uploading and integration, facilitating real-time access and analysis by clinicians. (3) For different clinical scenarios, implementation strategies should be differentially designed. For instance, ICU settings may deploy high-precision continuous monitoring devices to meet critical patient needs, while general wards may adopt intermittent monitoring solutions to balance resources and efficiency, thereby enhancing system versatility. Moreover, at the algorithmic level, privacy-preserving federated learning frameworks should be implemented to adapt the system to regional healthcare disparities (e.g., accessibility of anticoagulants in urban vs. rural hospitals). (4) Finally, a prediction-intervention feedback loop must be established, leveraging electronic health record systems to automatically track intervention outcomes (e.g., D-dimer decline rates after anticoagulation regimen escalation), enabling model self-evolution.

## 5 Conclusion

This study establishes an explainable early warning model that integrates improved intelligent algorithms with an AutoML framework, effectively predicting composite endpoint events after adolescent tibial fracture surgery. The model identifies key clinical features such as age, surgery duration, and FIB, along with their interaction effects, providing new perspectives for risk mechanism analysis. SHAP-based visualization techniques reveal key pathological processes such as synergistic damage from age-surgery duration and the FIB-CRP cascade, supporting transparent clinical decision-making. Although the three-tier warning system has not been validated in real environments, its stepwise management framework provides a feasible pathway for individualized interventions. With the advancement of prospective multicenter studies and the development of dynamic monitoring technologies, this system is expected to optimize adolescent fracture rehabilitation management practices and enhance surgical safety margins.

## Data Availability

The raw data supporting the conclusions of this article will be made available by the authors, without undue reservation.
