# Supplementary material for: Explainable machine learning-based prediction of early and mid-term postoperative complications in adolescent tibial fractures
Source: Front Surg. 2025 Oct 21;12:1688702. doi: 10.3389/fsurg.2025.1688702 (PMC12584154; doi:10.3389/fsurg.2025.1688702)
Supplement: Supplementary file 1 [file Table1.docx]

**Supplementary Material**

****Table S1: Complete Feature Preprocessing Specifications****

| Variable Name | Variable Type | Unit/Definition | Missing Rate | Processing Method |
| --- | --- | --- | --- | --- |
| Age | Continuous | Years | 0.00% | Z-score standardization |
| Sex | Binary | Male=1, Female=0 | 0.00% | Direct encoding |
| BMI | Continuous | kg/m² | 0.20% | Multiple imputation + Z-scoring |
| AO Classification | Ordinal | A=Simple(0), B=Wedge(1), C=Complex(2) | 0.00% | Ordinal encoding |
| Injury Mechanism | Multiclass | Sports/Traffic/Fall≥3m//Other | 0.30% | Mode imputation + One-Hot encoding |
| Operation Duration | Continuous | Minutes | 0.00% | Z-score standardization |
| Blood Loss | Continuous | mL | 0.10% | Z-score standardization |
| PT | Continuous | Seconds | 2.10% | Multiple imputation + Z-scoring |
| APTT | Continuous | Seconds | 2.40% | Multiple imputation + Z-scoring |
| Fibrinogen | Continuous | g/L | 1.90% | Multiple imputation + Z-scoring |
| D-dimer | Continuous | mg/L FEU | 2.90% | Natural log-transform + Multiple imputation + Standardization |
| CRP | Continuous | mg/L | 1.80% | Natural log-transform + Multiple imputation + Standardization |
| Smoking History | Binary | Yes=1, No=0 | 0.40% | Mode imputation |
